# Utilizing the Intrinsic Mode of Weakly Coupled Resonators for Temperature Compensation

**DOI:** 10.3390/mi13091447

**Published:** 2022-09-01

**Authors:** Kunfeng Wang, Xingyin Xiong, Zheng Wang, Pengcheng Cai, Liangbo Ma, Xudong Zou

**Affiliations:** 1 State Key Laboratory of Transducer Technology, Aerospace Information Research Institute, Chinese Academy of Sciences, Beijing 100190, China; 2 School of Electronic, Electrical and Communication Engineering, University of Chinese Academy of Sciences, Beijing 100049, China; 3 QiLu Aerospace Information Research Institute, Jinan 250101, China

**Keywords:** MEMS, mode localization, accelerometer, temperature compensation

## Abstract

Accelerometers based on outputting amplitude ratios in weakly coupled resonators (WCRs) are attractive because their parametric sensitivity is higher by two or three orders of magnitudes than those based on outputting frequency. However, the impact of temperature on the coupler is a key factor in accelerometer applications. This paper proposed a novel mode-localized WCR accelerometer with a temperature compensation mechanism, with sensitive elements incorporating a double-ended tuning fork (DETF) resonator, clamped–clamped (CC) resonator, and a micro-lever coupler. The DETF out-of-phase mode is utilized, which is only sensitive to temperature, to measure the temperature change of WCRs and complete the temperature compensation using the compensation algorithm. This proposed method has no time delay in measuring the temperature of sensitive elements and no temperature difference caused by the uneven temperature field. The parametric sensitivity in amplitude ratio (AR) to acceleration drifting with temperature was theoretically analyzed, and the novel device was designed and fabricated by a silicon-on-glass process. Both simulation and experiment results demonstrated that the coupling stiffness drifted with temperature, which resulted in the drifts of its sensitivity to acceleration and zero-bias stability. Using the intrinsic mode of WCRs, in terms of the DETF out-of-phase mode, as an in situ thermometer and carrying out the temperature compensation algorithm, the drift of zero bias could be suppressed from 102 mg to 4.5 mg (g is the gravity acceleration), and the drift of the parameter sensitivity in AR was suppressed from 0.74 AR/g to 0.02 AR/g with the temperature range from 330 K to 370 K and acceleration range from 0 g to 0.2 g.

## 1. Introduction

MEMS (microelectromechanical systems) accelerometers are widely used in the field of inertial navigation, medical consumer electronics, and automotives due to the merits associated with the compacted IC (integrated circuit) fabrication, small size, and low cost [1,2,3]. Their sensing principle consists of piezoelectric accelerometers [4], piezoresistive accelerometers [5], capacitive accelerometers [6], resonant accelerometers [1], and so on. Compared to them, resonant accelerometers have a better capability of high sensitivity and high stability, and a large dynamic range [1,7]. Recently, the mode localization effect in weakly coupled resonators (WCRs) has attracted researchers due to orders of magnitude enhancement in parametric sensitivity and its intrinsic common mode rejection [8,9]. Furthermore, the mode localization effect in WCRs has been used in sensing applications such as voltmeters [10], tilt sensors [11], mass sensors [12,13,14], and accelerometers [7,15,16].

WCRs couple identical resonators with a spring much weaker than that of the resonators. By weakening the coupling structure, parametric sensitivity can be improved [8]. Electrical coupling and mechanical coupling are two widely used methods to lower the coupling stiffness. Electrical coupling resonators can produce much lower coupling stiffness and control the coupling voltage to adjust the coupling stiffness [15,17]. However, the coupling voltage will induce noise, resulting in the deterioration of sensor resolution. Moreover, a low-noise highly stable tunable voltage reference is difficult in practical applications. Mechanical coupling structures for low coupling stiffness need well-designed sizes or geometries. Several researchers have designed mechanical weakly coupling resonant accelerometers [18,19]. Moreover, the parametric modulation method has been used to further improve the parametric sensitivity in the mechanical weakly coupling resonator [16].

The effective symmetrical resonators of mode-localized WCR accelerometers can be immune to the effect of the temperature variations thanks to its merit of the common mode rejection. However, mechanical coupling structures still suffer from the effects of temperature variations, Milind [20] and Kang [21] reported the effects of temperature on the amplitude ratio sensitivity of mechanically coupled and electrostatically coupled structures. Thus, temperature variation will cause the drift of the coupling stiffness and worsen the sensor’s stability, resolution, and sensitivity. So far as is known to the authors, this has not been investigated in detail. The temperature drift of the coupling stiffness comes from the Young’s modulus and thermal elastic stress of material [22,23]. To address this problem, several technical approaches have been provided in other types of accelerometers. They can be divided into two types, namely, actively controlled temperature [24,25,26,27], and passive temperature compensation, by software algorithms [28,29], structure design [3,22,30], or electronic circuitry [31,32,33]. Actively controlled temperature schemes achieve stable temperatures in the vicinity of the sensitive elements through the oven-cavity [24,25,26,27], which will improve the stability of the sensors, while sophisticated system design has higher power consumption, and it will need a long warm-up start time. One of the passive temperature compensation methods concerns the measurement of temperature by using a calibrated resonator or temperature-sensitive element [28,29]; then the compensation algorithm solves the acceleration by obtaining the temperature change. Moreover, some researchers have proposed differential structures of resonant accelerometer with differential frequencies shifts as its output metrics. In this way, the effects of temperature variation are suppressed due to common mode interference [3]. Another passive temperature compensation method is to directly use the circuit to compensate for temperature drifts, such as the electrostatic stiffness control output resonator output [33], then differential frequency modulation, and demodulation continuous temperature compensation [31]. The advantage of passive temperature compensation is that it does not have a complicated structure design, but fabricating the two identical resonators is difficult due to manufacturing tolerances. Although Prad and Zhao [2] have reported that the initial mismatch in stiffness can be tuned effectively via voltage control, this cannot suppress the effect of temperature on the sensitivity due to the presence of initial asymmetry and temperature characteristics of the coupling structure.

In this paper, we proposed a novel type of mode-localized accelerometer with a temperature compensation mechanism. The temperature drift of the coupling stiffness and its effects were investigated and characterized. The WCRs of the novel device consisted of a double-ended tune fork (DETF) resonator, a clamp–clamp (CC) resonator, and a micro-lever coupler (DETF-CC WCRs). The DETF in-phase mode was coupled with the CC resonator through a micro-lever coupler structure that produces mode localization effects when acceleration is induced. The DETF out-of-phase mode frequency is linearly sensitive to temperature change and insensitive to the acceleration that was verified with simulation and experiment. This method of temperature measurement has no time delay by using the resonator’s intrinsic mode and does not exit the temperature difference because the temperatures are obtained by the WCRs themselves. Then the accelerations can be compensated by measuring the temperature in combination with the temperature compensation algorithm. Based on the temperature compensation method, the parametric sensitivity in amplitude ratio (AR) and zero bias can be optimized 37 times and 22.7 times, respectively, in the temperature range of 330 K to 370 K and acceleration range of 0 g to 0.2 g.

This paper is organized as follows: Section 2 presents the theory analysis for the drift of the parametric sensitivity in AR of the mode-localized accelerometer due to temperature variation, the structure design of the mode-localized accelerometer with temperature compensation, and finite element multiphysics (FEM) simulation and compensation mechanism algorithm implementation. The experimental setup, test results, and discussion are demonstrated in Section 3. Finally, this paper is concluded in Section 4.

## 2. Theory Analysis and Method of Temperature Compensation

### 2.1. Structure Design

**Figure 1:**
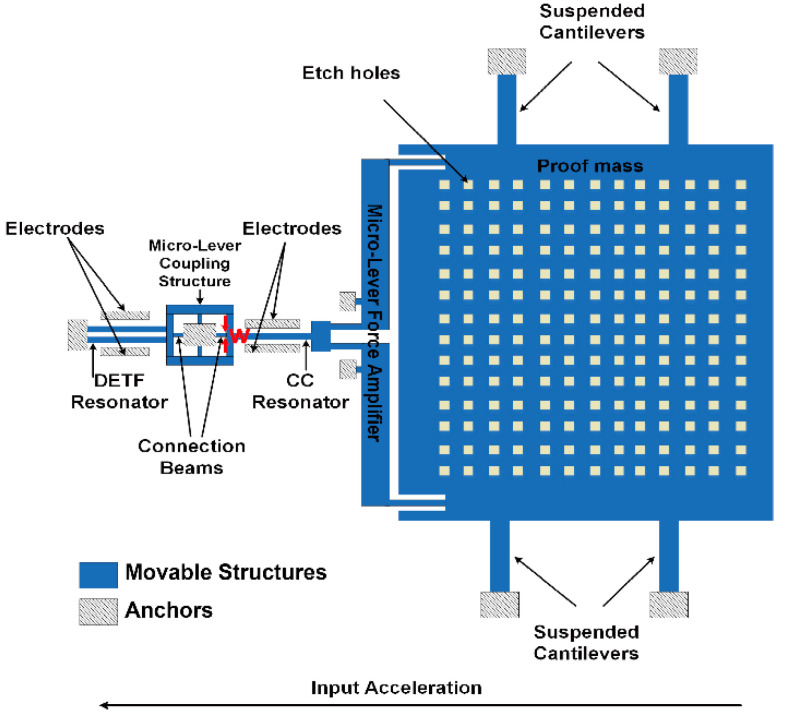
The schematic of the DETF-CC WCRs mode-localized accelerometer with micro-lever coupler structure.

The schematic of the mode-localized accelerometer is shown in Figure 1. The DETF resonator and CC resonator are coupled by a micro-lever coupler structure, the micro-lever coupler is designed to transfer the strain energy flow “leaked” from the non-ideal anchors of the resonators to enable mechanical coupling, and the width of connection beams (w) of the micro-lever coupling structure can be utilized to adjust the coupling strength of the DETF-CC WCRs system [18], increasing the dimensions of connection beams (w) of the micro-lever coupler for lower coupling stiffness, i.e., larger parametric sensitivity in AR.

The WCRs’ two resonators are driven and sensed by parallel-plate capacitors at two sides of the resonators. The proof mass is suspended by four cantilevers and through a pair of micro-lever force amplifiers connected to the CC resonator. The inertial force induced by acceleration is axially applied to the CC resonator. The axial force perturbs the effective stiffness of the CC resonator, breaking the initial symmetry state, which in turn induces the mode localization effect. Moreover, this stiffness perturbation will be treated as common-mode interference and will be canceled out when the DETF resonator vibrates in out-of-phase, i.e., the two beams of DETF resonator reverse vibration. This means the coupling energy transferred in DETF-CC WCRs is “blocked” when the DETF vibrates in the out-of-phase mode. Thus, the frequency of DETF’s out-of-phase is insensitive to acceleration variations and can be used as an in situ temperature sensor.

### 2.2. Theory Analysis

**Figure 2:**
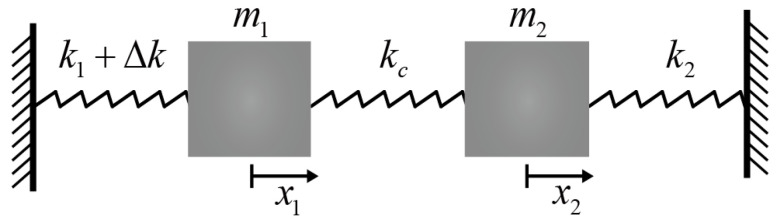
Lumped spring-mass model of the 2-DoF weakly coupled resonators.

To understand the temperature characteristics of the mode-localized accelerometer, a lumped model of the mode-localized accelerometer based on 2-DoF weakly coupled resonators is shown in Figure 2; the system is connected with a coupling spring ($k_{c}$). In the ideal case, we assume a system without damping and external forces, and the two-resonator structure parameters are assumed to be identical (i.e., $m_{1}=m_{2}=m$, and $k_{1}=k_{2}=k$ represent the effective mass and stiffness of the two resonators, respectively). The motion equations of the system can be given by

(1) $$\left[\begin{array}{cc} m & 0\\ 0 & m \end{array}\right]\left[\begin{array}{c} {\ddot{x}}_{1}\\ {\ddot{x}}_{2} \end{array}\right]+\left[\begin{array}{cc} k+k_{c} & -k_{c}\\ -k_{c} & k+k_{c}+\Delta k \end{array}\right]\left[\begin{array}{c} x_{1}\\ x_{2} \end{array}\right]=\left[\begin{array}{c} 0\\ 0 \end{array}\right]$$

where $x_{1}$ and $x_{2}$ represent the displacement of each resonator, and Δk is the stiffness perturbation.

Solving Equation (1), the resonant frequencies $\omega_{i}$ and the amplitude ratios $u_{i}$ (i=1,2) of the two vibrating modes can be derived as [34]

(2) $$\omega_{i}^{2}=\frac{2(k_{1}+k_{c})+\Delta k\mp \sqrt{\Delta k^{2}+4k_{c}^{2}}}{2m}$$

(3) $$u_{i}=\frac{x_{i1}}{x_{i2}}=\frac{\Delta k\pm \sqrt{\Delta k^{2}+4k_{c}^{2}}}{2k_{c}}$$

The coupling spring stiffness $k_{c}$ is affected by temperature; here, $k_{c}\ll k$, because Young’s modulus and the thermal expansion coefficient of the material are dependent on temperature. Thus, temperature changes will cause variations of mode-localized accelerometer output metrics, as seen in Equations (2) and (3), and frequencies and amplitude ratios are related to coupling spring stiffness $k_{c}$. Considering the temperature impact, the amplitude ratio of the system at temperature T can be rewritten as

(4) $$u\left(T\right)=\frac{\Delta k\pm \sqrt{\Delta k^{2}+4k_{c}{\left(T\right)}^{2}}}{2k_{c}\left(T\right)}$$

where $k_{c}\left(T\right)$ represents the coupling spring stiffness at temperature T. Due to the common mode rejection of mode localization, the effect of temperature on stiffness perturbation Δk can be negligible compared to the effect of temperature on coupling stiffness $k_{c}$.

**Figure 3:**
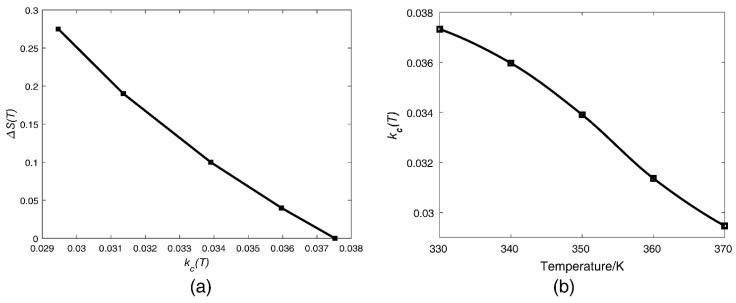
(**a**) The normalized drift of the parametric sensitivity in AR as with variation of coupling stiffness. Temperature ranges from 330 K to 370 K (these $k_{c}\left(T\right)$ data are extracted from simulation); (**b**) the coupling stiffness $k_{c}$ versus temperature T.

The amplitude ratio of the mode-localized accelerometer varies with temperature change due to the drift of $k_{c}$ with temperature. According to Equation (4), the parametric sensitivity in AR of the mode-localized accelerometer at temperature T can be derived as [35]

(5) $$S\left(T\right)=\frac{du(T)}{d(\Delta k)}\frac{d(\Delta k)}{da_{in}}=\gamma \frac{1\pm \frac{\Delta k}{\sqrt{\Delta k^{2}+4k_{c}{\left(T\right)}^{2}}}}{2k_{c}\left(T\right)}$$

where $a_{in}$ is loading accelerations, and γ is the ratio of stiffness perturbation to $a_{in}$. Equation (5) can be used to analyze the drift of the parametric sensitivity in AR due to temperature induction. The normalized drift of the parametric sensitivity in AR with temperature change can be expressed as

(6) $$\begin{array}{cc} \Delta S(T) & =\frac{S(T)-S(T_{0})}{S(T_{0})}=\frac{1\pm \frac{\Delta k}{\sqrt{\Delta k^{2}+4k_{c}{\left(T_{0}\right)}^{2}}}}{1\pm \frac{\Delta k}{\sqrt{\Delta k^{2}+4k_{c}{\left(T\right)}^{2}}}}\frac{k_{c}(T_{0})}{k_{c}(T)}-1\\ & \approx \left[\frac{k_{c}(T_{0})}{k_{c}(T)}\right]-1 \end{array}$$

where $k_{c}\left(T\right)=k_{c}\left(T_{0}\right)+\Delta k_{c}\left(T\right)$, $\Delta k_{c}\left(T\right)$ is the temperature-induced coupling stiffness variation compared with temperature $T_{0}$, and $k_{c}\left(T_{0}\right)$ and $k_{c}\left(T\right)$ are the coupling stiffness before and after temperature change, respectively. Equation (6) can be used to evaluate the temperature-induced coupling stiffness variation that causes the parametric sensitivity in AR ΔS(T) change (weaker coupling stiffness also follows the relationship of Equation (6)), as shown in Figure 3a. According to Equation (6) and Figure 3a, we can obtain the normalized drift of the parametric sensitivity in AR; ΔS(T) decreases with the increase of the coupling stiffness $k_{c}\left(T\right)$. Figure 3b shows the coupling stiffness $k_{c}\left(T\right)$ versus temperature *T*.

From Equations (5) and (6), it can be observed that the parametric sensitivity in AR depends on coupling stiffness $k_{c}$, which suggests the lower coupling stiffness will produce more dramatic mode localization effects when induced with the same stiffness perturbation. However, the impact of temperature (Young’s modulus or thermal expansion of material is dominant) on the coupling stiffness $k_{c}$ will cause the parametric sensitivity in AR change. That is one of the factors that hinders the further high-precision acceleration measurement of the weakly coupled resonators.

### 2.3. Simulation and Temperature Compensation

#### 2.3.1. Simulation of the Mode Localized Accelerometer Temperature Characteristic

**Figure 4:**
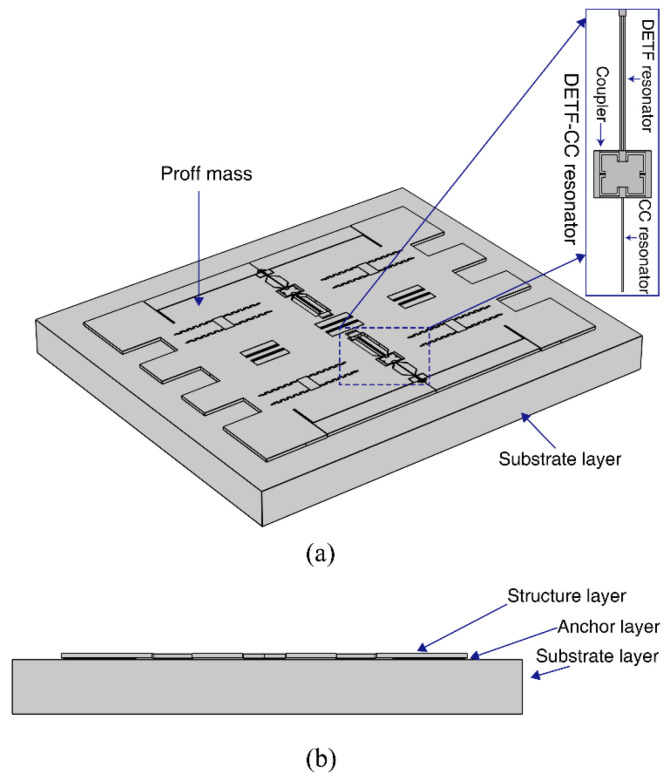
Simulation model of DETF-CC WCR mode-localized accelerometer with DETF-CC resonators (**a**) and left view (**b**).

**Figure 5:**
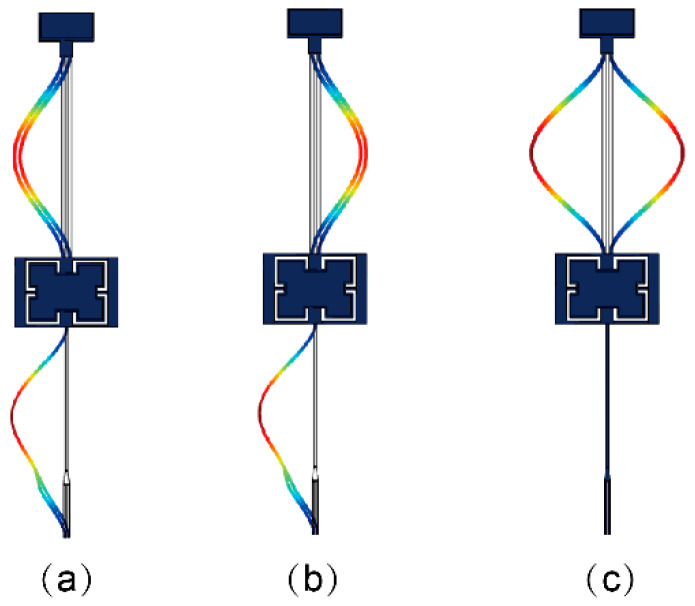
COMSOL simulation of three vibration modes of DETF-CC WCRs. (**a**) The WCRs in-phase mode; (**b**) the WCRs out-of-phase mode; (**c**) the DETF out-of-phase mode.

The temperature characteristic of the DETF-CC WCRs mode-localized accelerometer is simulated with the FEM simulation software COMSOL 5.4; the simulation model is shown in Figure 4. The model consists of a structure layer, an anchor layer, and a substrate layer; the material of the structure layer and the anchor layer is silicon; and the material of the substrate layer is Pyrex 7740 glass. The structure layer is fixed at the substrate layer with the anchors. These geometrical dimensions are summarized in Table 1. The three vibration modes of the DETF-CC WCRs are simulated, and the results are shown in Figure 5. The DETF resonator with the in-phase mode is weakly coupled with the CC resonator. The WCR has two vibration modes, which are defined as the in-phase mode of WCR and the out-of-phase mode of WCR, as shown in Figure 5a,b, respectively. The DETF in-phase mode coupling with the CC resonator through the micro-lever coupler structure produces a mode localization effect. When the perturbation from acceleration is subjected to the CC resonator, the effective stiffness of the CC resonator will be changed, whereas the effective stiffness of the DETF resonator will be unchanged, then breaking the initial symmetry and causing the effect of mode localization. The DETF resonator with the out-of-phase mode “blocks” the coupling with the CC resonator. The vibration mode is defined as out-of-phase of DETF, as shown in Figure 5c.

**Figure 6:**
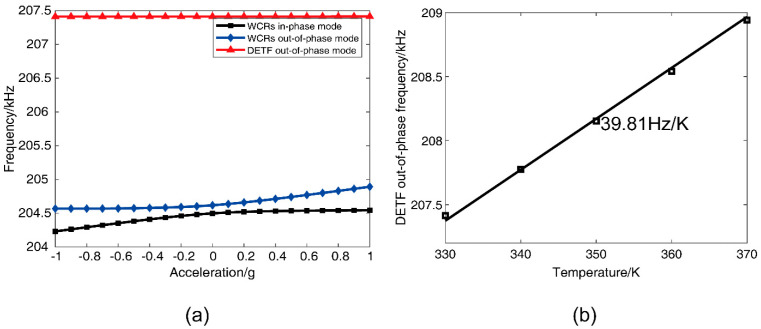
(**a**) The simulation results of the DETF-CC WCRs three mode frequency shift with the acceleration from –1 g to +1 g (the effective perturbation stiffness δk=Δk/k is from −0.005 to +0.005); (**b**) the frequency of DETF out-of-phase versus temperature at 0 g.

The corresponding frequency shifts of the three modes in DETF-CC WCRs with acceleration variations from −1 g to +1 g at 330 K temperature are shown in Figure 6a. The frequency shift of the DETF out-of-phase mode is 10 Hz (24 ppm/g), as shown in the red curve in Figure 6a. Its small frequency shift verifies that this mode is insensitive to acceleration change. The frequency shifts of the other two modes demonstrate the typical mode-localization effect resulting from the stiffness perturbation induced by acceleration, and the frequency shifts are both 320 Hz, as shown in the black curve and the blue curve of Figure 6a, respectively. Figure 6b shows that the DETF’s out-of-phase mode frequency increases linearly with a temperature increase, and the temperature coefficient is 39.81 Hz/K. Thus, according to the linear relationship, this mode frequency can be viewed as a thermometer that reflects the temperature variations of the WCRs. According to Equation (5), we can know that the parametric sensitivity in AR increases by weakening the coupling stiffness $k_{c}\left(T\right)$. The coupling stiffness $k_{c}\left(T\right)$ can be calculated from the mode of the frequency difference of the veering zone as with acceleration at different temperatures [36]. It is shown in Figure 3b that the variation of coupling stiffness $k_{c}\left(T\right)$ with the temperature ranges from 330 K to 370 K, and the increase in temperature will cause a decrease in the coupling stiffness $k_{c}\left(T\right)$. Thus, the increase in temperature will cause an increase in the parametric sensitivity in AR.

### 2.3.2. Temperature Compensation Mechanism and Algorithm Implementation

**Figure 7:**
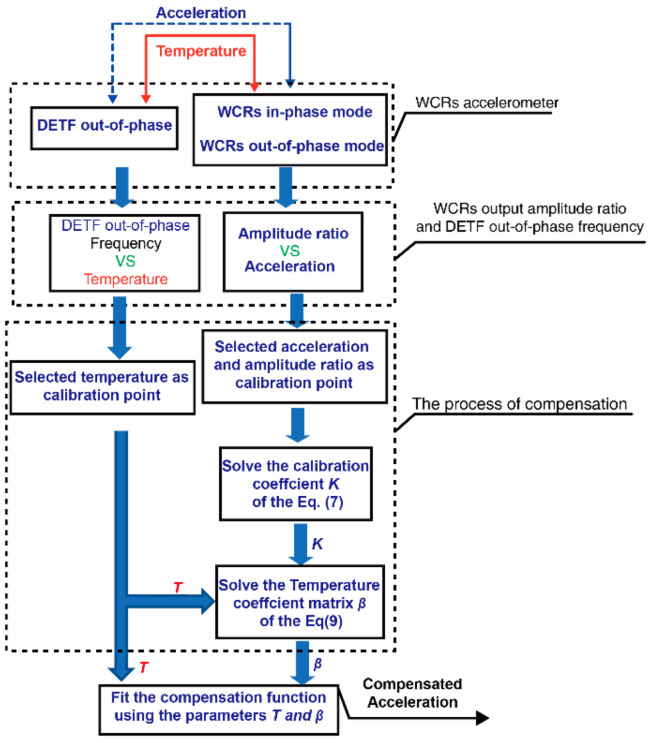
The flow charts implement the DETF-CC WCRs accelerometer temperature compensation method.

The simulation results illustrate that the DETF out-of-phase mode can be viewed as a thermometer, and then according to the measured temperature change from DETF out-of-phase mode frequency to compensate for the acceleration, a detailed process of the temperature compensation method is shown in Figure 7.

In the first step, the DETF-CC WCR accelerometer is subjected to temperature and acceleration, rhe WCRs in-phase mode and WCRs out-of-phase mode output amplitude ratio, and the DETF out-of-phase mode outputs frequency. Then, the amplitude ratio of the WCRs in-phase mode and WCRs out-of-phase mode is measured, and the DETF out-of-phase mode frequency at different temperature-loaded accelerations is collected; fitting the relationship between temperature and DETF out-of-phase mode frequency, we can obtain the temperature of WCRs from the DETF out-of-phase mode frequency during the compensation process. The next step is to select the calibration points about the amplitude ratios and accelerations versus different selected temperature calibration points, which can be expressed as

(7) $$\left[\begin{array}{c} AR(T_{0})\\ AR(T_{1})\\ \vdots \\ AR(T_{m}) \end{array}\right]=\left[\begin{array}{cccc} k_{00} & k_{01} & \cdots & k_{0n}\\ k_{10} & k_{11} & \cdots & k_{1n}\\ \vdots & \vdots & \ddots & \vdots \\ k_{m0} & k_{m1} & \cdots & k_{mn} \end{array}\right]\left[\begin{array}{c} 1\\ a\\ \vdots \\ a^{n} \end{array}\right]$$

where AR(T) is selected for m temperature calibration points of the amplitude ratios of the mode-localized accelerometer output, a is the loaded accelerations, and $k_{mn}$ is the temperature-dependent calibration coefficient. Substituting the selected temperature calibration points of the amplitude ratios into Equation (7), the obtained $k_{mn}$ can be written as

(8) $$K=\left[\begin{array}{cccc} K_{0} & K_{1} & \cdots & K_{n} \end{array}\right]$$

where $K_{n}={\left[\begin{array}{cccc} k_{0n} & k_{1n} & \cdots & k_{mn} \end{array}\right]}^{⊤}$, the ***K*** is a temperature-dependent function, and the relationship between ***K*** and temperature *T* can be expressed as

(9) $$\left[\begin{array}{c} k_{0n}\\ k_{1n}\\ \vdots \\ k_{mn} \end{array}\right]=\left[\begin{array}{cccc} \beta_{00} & \beta_{01} & \cdots & \beta_{0q}\\ \beta_{10} & \beta_{11} & \cdots & \beta_{1q}\\ \vdots & \vdots & \ddots & \vdots \\ \beta_{p0} & \beta_{p1} & \cdots & \beta_{pq} \end{array}\right]\left[\begin{array}{c} 1\\ T\\ \vdots \\ T^{q} \end{array}\right]$$

where (p=0, 1, 2⋯m); solving Equation (9), we can obtain the temperature calibration coefficient matrix ***β***.

Finally, the compensation function is fit using the temperature calibration coefficient matrix ***β*** and temperature *T*. Substituting Equation (9) into Equation (7), the relationship between the amplitude ratios and accelerations about temperature can be expressed as

(10) $$AR(T_{m})={\left(\left[\begin{array}{cccc} \beta_{00} & \beta_{01} & \cdots & \beta_{0q}\\ \beta_{10} & \beta_{11} & \cdots & \beta_{1q}\\ \vdots & \vdots & \ddots & \vdots \\ \beta_{p0} & \beta_{p1} & \cdots & \beta_{pq} \end{array}\right]\left[\begin{array}{c} 1\\ T\\ \vdots \\ T^{q} \end{array}\right]\right)}^{⊤}\left[\begin{array}{c} 1\\ a\\ \vdots \\ a^{p} \end{array}\right]$$

**Figure 8:**
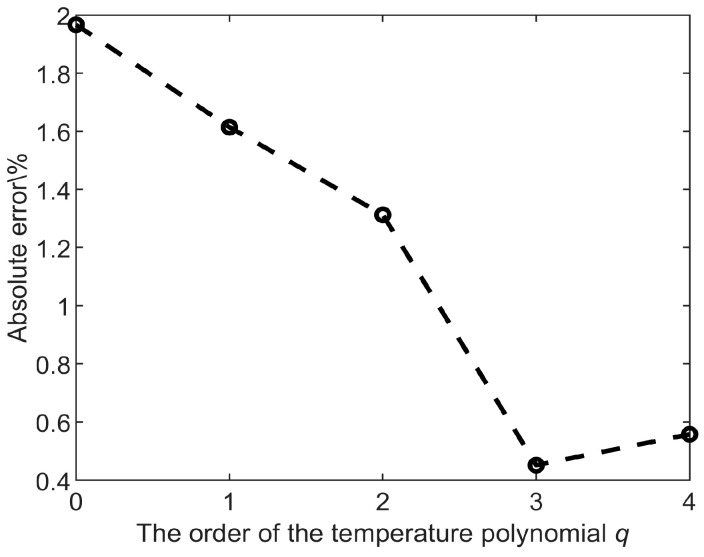
The compensation absolute errors are influenced by the fitting order *q*, when *q* = 3 is the optimal order of temperature polynomial *q*.

The compensation accelerations can be calculated with the compensation function (Equation (10)) by post-processing (such as the least square method using MATLAB or a digital circuit). Here we choose *n = p =* 1 due to the linear relationship between acceleration a and amplitude ratio AR. The choice of the degree of the temperature polynomial *q* is significant for the compensation accuracy because the parametric sensitivity in AR is not a linear relationship with temperature. The optimized results are shown in Figure 8; in the range of 330 K to 370 K, when *q* was increased from 0 to 3, a decrease of the absolute error after temperature compensation was remarked. Further increasing *q*, the compensation accuracy becomes worse due to overfitting. According to these results, the optimized degree of the temperature polynomial is *q* = 3, and the compensation absolute error is minimal.

## 3. Experimental Results

### 3.1. Experimental Setup

**Figure 9:**
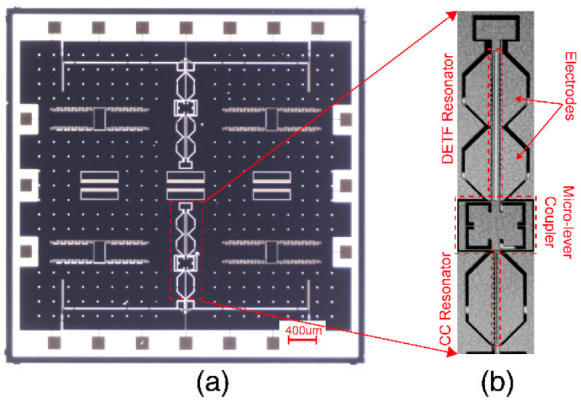
The optical micrographs of the mode-localized accelerometer: (**a**) overview of the mode-localized accelerometer; (**b**) details of the DETF resonator, CC resonator, and micro-lever coupler.

**Figure 10:**
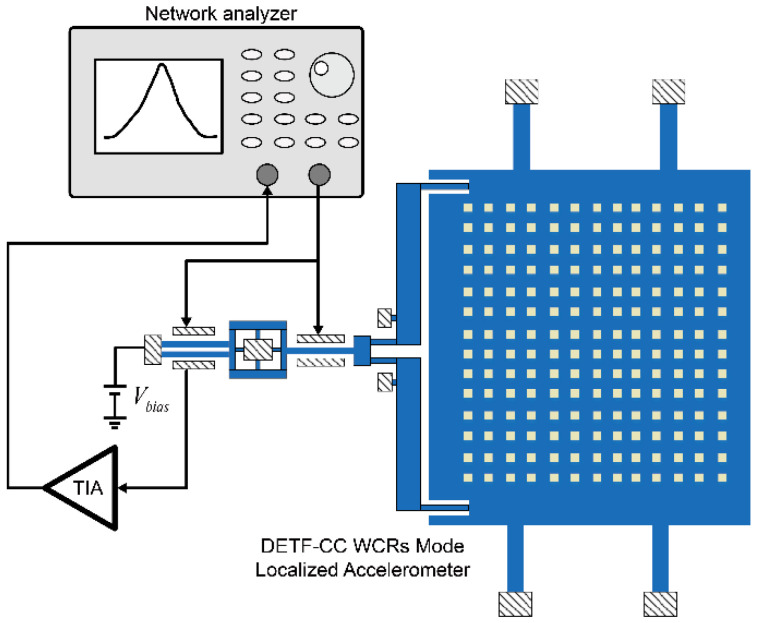
Open-loop experimental setup of the DETF-CC accelerometer.

The prototype devices (geometry dimensions are shown in Table 1) of the DETF-CC WCRs mode-localized accelerometer were fabricated by the silicon-on-glass (SOG) foundry process. The optical micrographs and structure detail view of the DETF-CC WCRs are shown in Figure 9. The DC bias voltage $V_{bias}$ of 10 V was applied to the resonators, the AC drive sweep signal ($V_{AC}$ is 9 mV to ensure that the resonators work in the linear operation) was applied to the drive electrodes to excite resonators, and the sense electrode connected a transimpedance amplifier (TIA) for transforming the motional current to voltage, and voltage feedback to the receiver port of the network analyzer (Keysight E5061B), as shown in Figure 10. The amplitude–frequency responses were recorded with the network analyzer. The temperature control was realized by placing the device in the Lakeshore probe station with a vacuum chamber and a Model 336 temperature controller.

### 3.2. Experimental Results

#### 3.2.1. Mode Analysis and Sensitivity

**Figure 11:**
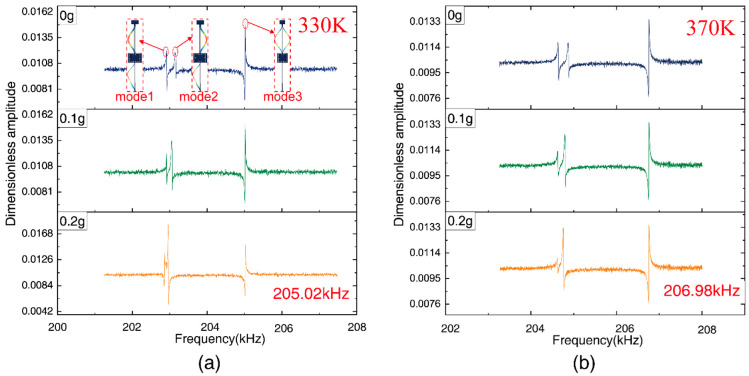
Amplitude–frequency response of the mode-localized accelerometer of 330 K (**a**) and 370 K (**b**) with different accelerations (0 g–0.2 g).

**Figure 12:**
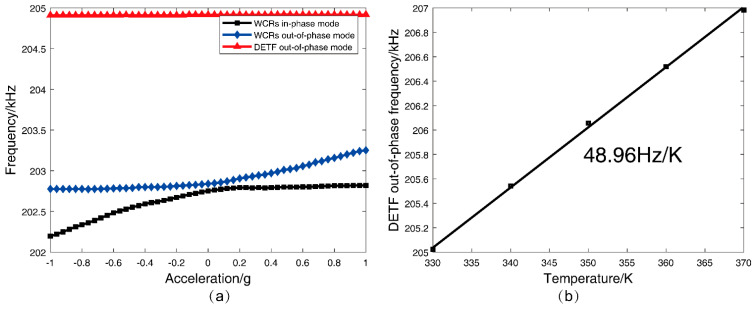
(**a**) The experimental results of the DETF-CC WCRs three mode frequency shift with the acceleration from –1 g to +1 g; (**b**) the frequency of DETFs out-of-phase versus temperature at 0 g.

For the DETF-CC WCRs, there were three vibration modes, as shown in Figure 5. The amplitude–frequency response curves of all the three vibration modes are plotted in Figure 11. Mode 1 is the WCRs in-phase mode, mode 2 is WCRs out-of-phase mode, and mode 3 is DETF out-of-phase mode, with respect to Figure 5a–c. Figure 11 shows the amplitude–frequency responses curves of the DETF-CC WCRs mode-localized accelerometer when subjected to the accelerations of 0 g, 0.1 g, and 0.2 g. Figure 11a depicts the amplitude–frequency responses curves at 330 K, and Figure 11b corresponds to measurements at 370 K. It can be observed from Figure 11 that the mode 1 frequency increased with the acceleration, and the mode 2 frequency remained constant. The amplitude of the two modes changed drastically with the acceleration increases due to mode localization effect. Moreover, the frequency and amplitude of mode 3 were nearly constant when acceleration changed at the same temperature. The frequency of mode 3 drifted upward with temperature increases (205,023.12 Hz at 330 K drifted to 206,982.59 Hz at 370 K), which verified that mode 3 is insensitive to accelerations and can be used as a temperature sensor. Figure 12a shows the experimental results of the frequency shift of the DETF-CC WCRs modes with an acceleration range from −1 g to +1 g at 330 K temperature, and a frequency shift of the DETF out-of-phase mode (mode 3) of 8.73 Hz (it is 21.52 ppm/g); its shift could be neglected compared with the two work modes variation. The DETF-CC WCRs temperature changed from 330 K to 370 K, the DETF out-of-phase mode frequency (mode 3) was linearly related to the temperature, and the temperature coefficient was 48.96 Hz/K, as shown in Figure 12b. The measured mode frequency was larger than the simulation due to the fabrication process tolerance. The temperature coefficient of mode 3 was larger than the simulation, perhaps because of the residual stress [37].

**Figure 13:**
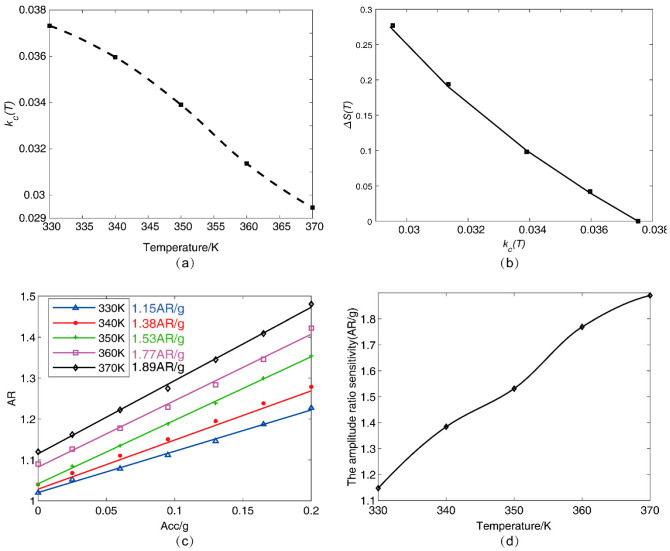
(**a**) The experimental results of the coupling stiffness $k_{c}$ versus temperature T, and (**b**) the experimental results of the normalized drift of the parameter sensitivity in AR with variation of the coupling stiffness with temperature ranges from 330 K to 370 K; (**c**) the amplitude ratio of the mode-localized accelerometer at different temperatures ranging from 330 K to 370 K in 10 K steps; (**d**) temperature coefficient of accelerometer output amplitude ratio range from 0 g to 0.21 g.

Figure 13 plots the experimental results on how temperature affects the coupling stiffness $k_{c}$, which further affects the drift of parameter sensitivity in AR. Figure 13a depicts the coupling stiffness $k_{c}$ versus T, and Figure 13b depicts the normalized drift of the parametric sensitivity in AR ΔS(T) as with variation of coupling stiffness $k_{c}$, validating the theory and simulation results. To investigate whether the parameter sensitivity in AR will increase with the temperature increase, the amplitude ratios with input acceleration ranging from 0 g to 0.2 g were measured under different temperatures from 330 K to 370 K, as shown in Figure 13c. The amplitude ratios had clear variations at different temperatures under the same acceleration. The parameter sensitivity in AR of the DETF-CC WCRs mode-localized accelerometer was 1.15 AR/g at 330 K temperature. When the temperature increased to 370 K, the parameter sensitivity in AR of the DETF-CC WCRs mode-localized accelerometer increased to 1.89 AR/g. The parametric sensitivity in AR improved approximately two orders of magnitude over the parametric sensitivity in frequency shift (temperature range from 330 K to 370 K). The drift of the parameter sensitivity in AR was up to 0.74 AR/g in the temperature range from 330 K to 370 K due to the temperature change, which caused a decrease of coupling stiffness, as shown in Figure 13a. There was a positive correlation between temperature and parametric sensitivity in AR due to the thermal elastic stress effect, as shown in Figure 13d.

#### 3.2.2. Temperature Compensation Experimental Results

**Figure 14:**
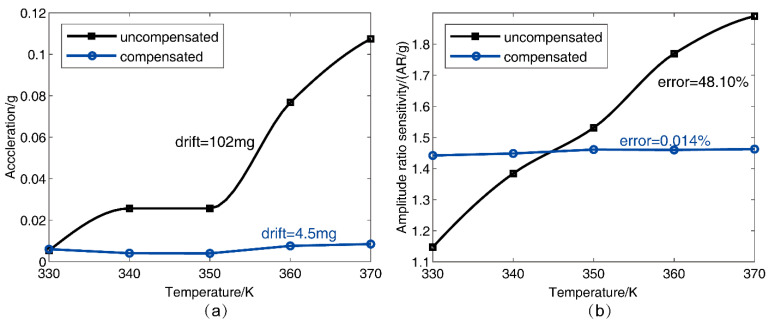
The experimental results of the accelerometer temperature compensation at the accelerometer of (**a**) zero bias 0 g, and (**b**) sensitivity.

Obtaining the corresponding temperature of the WCR accelerometer according to the DETF out-of-phase mode frequency change, the temperature was applied to solve the temperature calibration coefficient matrix β of Equation (9), substituting the temperature calibration coefficient matrix β into Equation (10) and then obtaining the accurate accelerations. Figure 14 shows that the zero bias and the parameter sensitivity in AR were compensated using the temperature compensation flow shown in Figure 7. The temperature changed from 330 K to 370 K at zero bias 0 g (Figure 14a) and parameter sensitivity in AR (Figure 14b), respectively. It can be seen that the temperature drifts of the WCR accelerometer were improved after compensation, and the zero bias drift was optimized from 102 mg to 4.5 mg at 0 g in the range from 330 K to 370 K, and the temperature coefficient of zero bias (abbreviated TCZB) was 0.11 mg/K; before compensation, the max error of the parameter sensitivity in AR was higher than 40.96% (the drift of the parameter sensitivity in AR was 0.74 AR/g), and the drift was lower than 0.014% (the drift of the parameter sensitivity in AR was suppressed to 0.02 AR/g) after applying the temperature compensation method in the range from 330 K to 370 K. Table 2 gives a brief performance comparison with the previously published accelerometer based on amplitude ratio and frequency, including 1-DoF, 2-DoF, and 3DoF. In our work, the normalized temperature drift of the sensitivity (abbreviated NTDS) could be suppressed by one order of magnitude lower than the previous works [30,37]. The TCZB could be optimized two times better than [37] and three times more than [38].

## 4. Conclusions

In this paper, a novel mode localization accelerometer with temperature compensation was proposed. The parameter sensitivity in AR to acceleration drift with temperature for mechanical WCRs was subjected to theoretical analysis. The DETF-CC WCR acceleration sensor was designed, simulated, fabricated, and experimentally measured. The intrinsic mode of DETF-CC WCRs, i.e., DETF out-of-phase mode, could be used as an in situ thermometer, which benefits from a zero delay and more accurate temperature measurement. By carrying out the temperature compensation algorithm after tested temperature characterization of the DETF-CC WCRs, both the temperature drift of the parameter sensitivity in AR and the temperature drift of zero bias could be suppressed by one order of magnitude lower than without temperature compensation.

In the future, a low noise temperature compensation circuit with mode switching to tackle the dynamic features of the novel device will be investigated. This device has the potential to achieve a high-performance accelerometer in terms of ultra-low temperature drift and high long-term stability. Moreover, it can apply other types of sensors based on mode localization effects, such as displacement sensor, charge sensor, etc.

## Figures and Tables

**Table 1:** Structure dimensions of the mode-localized accelerometer.

Parameters	Value
Total chip size	20 mm^3^
Structure thickness	40 μm
Anchor thickness	20 μm
Glass thickness	50 μm
Length of CC resonator	400 μm
Width of CC resonator	7 μm
Length of DETF resonator	600 μm
Width of DETF resonator	9.5 μm
Lever amplification ratio	23
Gap of parallel-plate capacitor	2 μm
Coupling stiffness at room temperature	0.037 N/m
The effective stiffness of CC resonator	335.23 N/m
The effective stiffness of DETF resonator	486.06 N/m
The effective mass of CC resonator	0.20 μg
The effective mass of DETF resonator	0.29 μg
Proof mass	1.5 mg

**Table 2:** Performance comparison.

	This Work	[21]	[31]	[38]
NTDS	0.014%	0.94%	0.53%	-
TCZB	0.11 mg/K	0.22 mg/K	-	263 K–343 K
Temperature Range	330 K–370 K	303 K–333 K	303 K–348 K	263 K–343 K
Degree-of- freedom	2-DoF	3-DoF	1-DoF	1-DoF
Output metric	Amplitude Ratio	Amplitude Ratio	Frequency	Frequency

## Data Availability

Not applicable.
